# Fibroblast Growth Factor-2 and the HIV-1 Tat Protein Synergize in Promoting Bcl-2 Expression and Preventing Endothelial Cell Apoptosis: Implications for the Pathogenesis of AIDS-Associated Kaposi's Sarcoma

**DOI:** 10.1155/2011/452729

**Published:** 2011-10-09

**Authors:** Cecilia Sgadari, Giovanni Barillari, Clelia Palladino, Stefania Bellino, Brunella Taddeo, Elena Toschi, Barbara Ensoli

**Affiliations:** ^1^National AIDS Center, Istituto Superiore di Sanità, 00161 Rome, Italy; ^2^Department of Experimental Medicine, University of Rome Tor Vergata, 00133 Rome, Italy; ^3^Marjorie B. Kovler Viral Oncology Laboratories, The University of Chicago, Chicago, IL 60637, USA

## Abstract

Kaposi's sarcoma (KS) is a vascular tumor frequently occurring in Human Immunodeficiency Virus- (HIV-) 1-infected individuals. Our previous work indicated that the angiogenic fibroblast growth factor (FGF)-2 and the Tat protein of HIV-1, both expressed in KS lesions of HIV-infected patients, synergize at inducing angioproliferative, KS-like lesions in mice. Here we show that the development of angioproliferative lesions promoted in mice by combined Tat and FGF-2 associates with an increase in the levels of expression of the antiapoptotic Bcl-2 protein. Upregulation of Bcl-2 expression by combined FGF-2 and Tat occurs also *in vitro*, and this protects human primary endothelial cells from programmed cell death. As Bcl-2 is expressed in human KS lesions in a fashion paralleling the progression of the disease, these findings suggest a molecular mechanism by which Tat and FGF-2 cooperate in KS maintenance and progression in HIV-infected individuals.

## 1. Introduction

KS arises as multiple patch, plaque, or nodular lesions, which are generally located on the skin or mucosas but can also involve visceral organs (reviewed in [1]). 

Early-stage KS lesions resemble an inflammatory, granulation-type reaction, and they are characterized by abnormal angiogenesis, leukocyte infiltration, endothelial cell activation, edema and by the proliferation of endothelial-like, spindle-shaped cells which are considered to be the KS tumor cells (KS cells) [1].

The development of KS lesions is triggered by inflammatory mediators and growth factors [1], whose production can be induced or enhanced by the human herpesvirus-8 (HHV-8), a viral agent infecting nearly all KS patients (reviewed in [2]). 

Among the growth factors expressed in KS lesions, FGF-2 is key for KS development. Specifically, upon its production by KS cells or infiltrating leukocytes, FGF-2 stimulates the locomotion and growth of both endothelial and KS cells by paracrine and autocrine mechanisms, respectively [1]. Consistently, FGF-2 can directly promote the development of KS-like lesions *in vivo* [3, 4], and it mediates the formation of angioproliferative lesions induced by KS cell injection in nude mice [5].

Despite rare and slowly progressive in general population, KS becomes frequent and aggressive in HIV-infected individuals (Acquired Immune Deficiency Syndrome- [AIDS-] associated KS) [1]. 

In this regard, previous results indicated that the Tat protein of HIV-1 can act as a progression factor in AIDS-KS. In particular, Tat, a transactivator of HIV-1 gene expression which is essential for virus replication, is released by HIV-1 acutely infected T cells (reviewed in [6]). By engaging cell surface receptors belonging to the integrin family, extracellular Tat induces endothelial or KS cell locomotion and adhesion [6]. At the same time, Tat competes FGF-2 binding to the heparan-sulphate proteoglycans of the cell surface and extracellular matrix (ECM) [7]. In doing so, Tat can retrieve sequestered FGF-2 into a soluble, biologically active form which, in turn, promotes endothelial or KS cell growth [7].

While early-stage KS lesions have a polyclonal nature and may regress, late nodular KS lesions can evolve into a true sarcoma [1]. Noteworthy, at this stage of disease progression, KS lesions undergo a rapid growth in the presence of a low mitotic index [8]. This suggested that vascular cell survival could be prevalent in KS maintenance and progression. 

In this context, the protein levels of Bcl-2, a potent antagonist of programmed cell death (apoptosis), were found to be increased in late-stage, as compared to early-stage, lesions from all forms of KS [8–12]. Coexpression of Bcl-2 and endothelial cell markers [9, 10] indicates that Bcl-2 is upregulated in activated endothelial cells lining the newly formed, abnormal blood vessels characterizing KS lesions. Consistently, Bcl-2 up-regulation is associated with the reduction or absence of endothelial cell apoptosis [8, 9].

At the present time, however, little or nothing is known about the mechanisms of Bcl-2 induction in KS.

Since either Tat or FGF-2 has been shown to modulate Bcl-2 expression by a wide variety of cell types [13–21], herein we evaluated the effects of FGF-2 and Tat, alone or combined, on Bcl-2 expression in animal and *in vitro* experimental models previously employed to study AIDS-KS pathogenesis.

## 2. Materials and Methods

### 2.1. Reagents

Recombinant HIV-1 Tat protein (from the IIIB isolate) was obtained, purified, tested for biological activity, and handled as previously described [22]. Human recombinant FGF-2 was purchased from Roche Molecular Biochemicals (Indianapolis, IN). Bovine serum albumin (BSA, fraction V), heparin (sodium salt, from porcine intestinal mucosa), gelatin (denatured collagen I, from bovine skin), and the chemicals employed for protein extraction were from Sigma (St. Louis, MO). Matrigel, a reconstituted basement membrane, and endothelial cell growth supplement (ECGS) were obtained from BD Bioscience (Bedford, MA). The phosphate buffered saline (PBS) solution, RPMI 1640 growth medium, and its supplements were from Invitrogen (Paisley, Scotland, UK).

### 2.2. Animal Studies

Recombinant FGF-2 and/or Tat protein (1 *μ*g and 10 *μ*g, resp.) were suspended in 0.2 mL of PBS-0.1% BSA, mixed with an equal volume of Matrigel and then injected subcutaneously into the lower back of Balb-c nu/nu female mice (4–6-week old, Charles River Breeding Laboratories, Calco, Italy), as previously described [3, 4]. Control animals were inoculated with the same volume of Matrigel and the buffer (PBS-0.1% BSA) employed to suspend FGF-2 or Tat. Seven days later, mice were sacrificed, and, at this time, the sites of injection were evaluated for the presence of macroscopic lesions [3, 4]. Tissue samples were taken and fixed in formalin or frozen in OCT compound (Miles Laboratories, Naperville, IL). Slides obtained from formalin-fixed paraffin-embedded blocks were examined at the microscope after hematoxylin-eosin staining. Frozen tissue sections were fixed in acetone and stained with 0.4 *μ*g/mL of anti-Bcl-2 rabbit polyclonal antibody (Santa Cruz Biotechnology, Santa Cruz, CA) by the peroxidase-anti-peroxidase method (Dako) [3, 4]. The percentage of positive cells was determined from the mean of 5 high-power fields (40X magnification) and expressed as the mean (minimum and maximal range) of values obtained. The care and use of mice were in accordance with the European Community guidelines. 

### 2.3. Cell Cultures

Human umbilical vein endothelial cells (HUVECs) were obtained from Lonza (Verviers, Belgium), seeded on surfaces precoated with gelatin, and cultured in RPMI 1640 medium supplemented with 15% fetal bovine serum (FBS), sodium pyruvate, L-glutamine, MEM essential and nonessential amino acids, heparin (1 *μ*g/mL), and ECGS (45 *μ*g/mL). Experiments were performed in the absence of ECGS or heparin.

### 2.4. Western Blot Analysis

At the end of each experiment, HUVECs were washed in ice-cold PBS, scraped off the flask, and lysed in 50 nM Tris pH 7.5, 1 mM phenylmethylsulfonyl fluoride (PMSF), 2 mM EGTA, 10 mM DTT, 5 *μ*g/mL aprotinin, 200 *μ*g/mL leupeptin, and 0.15% triton X 100. The protein content in the cell lysates was assayed with the Bradford reagent (Bio-Rad, Hercules,CA). Eight *μ*g of proteins from each experimental condition were subjected to 12% sodium dodecyl sulfate-polyacrylamide gel electrophoresis followed by transfer onto nitrocellulose membrane (Amersham Pharmacia Biotech, Little Chalfont, Buckinghamshire, UK). Filters were rinsed in blocking buffer (5% nonfat dry milk, 0.1% Tween-20 PBS), probed with anti-Bcl-2 monoclonal antibodies (2 *μ*g/mL, Santa Cruz), incubated with horseradish peroxidase-conjugated goat anti-mouse secondary antibodies (Amersham), and developed with the use of the enhanced chemiluminescence (ECL) method (Amersham). Intensity of the Bcl-2-specific band was quantified by densitometry, using an Imaging Densitometer GS-700 and a Multi-Analyst software (Bio-Rad). To verify equal loading of protein in each lane, blots were reprobed with anti-*β* actin monoclonal antibodies (Sigma). 

### 2.5. Detection of Apoptosis

HUVECs were seeded on plates coated with gelatin or with 10 *μ*g/mL of poly-2-hydroxymethyl-methacrylate [poly(HEMA)] [23]. Cells were exposed to FGF-2, Tat, or their suspension buffer and then collected. The entity of cell death was determined by using two different assays. 

The first was the Programmed Cell Death ELISA kit (Roche) which measures the amount of nucleosomes released in the cytoplasm of dying cells. Briefly, HUVECs were lysed, and the cytoplasmic fraction was recovered after centrifugation, and nucleosomes assayed as indicated by the manufacturer. Each sample was tested in duplicate. 

The second method was the terminal deoxynucleotidyl transferase-mediated dUTP nick end labeling (TUNEL) assay. Briefly, HUVECs were spun on slides by cytospin, fixed in 4% paraformaldehyde, and the DNA strand breaks of apoptotic cells were identified in situ by TUNEL kit (Roche), according to the manufacturer's instructions. Fluoresceinated nucleotides incorporated in polymers by the terminal deoxynucleotidyl transferase-based enzymatic reaction were detected by immuno-histochemical staining using a mouse antifluorescein isothiocyanate (FITC) monoclonal antibody and the alkaline phosphatase-anti-alkaline phosphatase method (Dako) [3]. The percentage of positive, apoptotic cells was determined from the mean of 4 high-power fields. 

### 2.6. Reverse Transcriptase-Polymerase Chain Reaction (RT-PCR)

Total RNA was extracted from HUVECs with the RNA assay Mini kit (Qiagen, Hilden, Germany) and further purified by three-round digestion with pancreatic DNAse I (10 IU/sample). Purified RNA (0.5 *μ*g) was retrotranscribed with the reverse transcription system kit (Promega, Madison, WI, USA) by incubating the reactions with hexanucleotide random primers for 10 min at room temperature, 30 min at 42°C, and 30 min at 53°C. After heat inactivation of the RT reaction, each sample was subjected to a low (pre-plateau) number of cycles of PCR (28 to 30) for *β*-actin with primer BA-1 [5′-CATGTGCAAGGCCGGCTTCG-3′, nucleotide (nt) 1137 to 1156], located in the first exon of the human *β*-actin gene, and primer BA-4 (5′-GAAGGTGTGGTGCCAGATTT-3′, nt 1475 to 1494) designed in the second exon of the gene (nt enumeration as in GenBank M10277). These primers yield a 226 bp cDNA amplicon; contaminant DNA is revealed by the appearance of a 357 bp genomic amplicon. The amount of cDNA to be used for Bcl-2 PCR was determined after cDNA normalization by pre-plateau *β*-actin PCR. The normalized samples were then subjected to 35 cycles of PCR for Bcl-2. Bcl-2 primers were 5′-TCGCCCTGGTGGACAACA-3′ (nt 1957 to 1975) and 5′-TGACTTCACTTGTGGCTCAGA-3′ (nt 2183 to 2163) (nt enumeration as in GenBank M13994). Conditions for Bcl-2 amplification were as follows: 4 min at 94°C, followed by 35 cycles of denaturation at 94°C for 1.5 min, annealing at 58°C for 1.5 min, and extension at 72°C for 2 min. Negative control reactions lacking DNA template were always performed in parallel to control for sample to sample contamination.

PCR-amplified products were separated onto a 1.8% agarose gel, blotted on nylon membrane, and hybridized to a 32 [P]-labeled oligonucleotide probe internal to the amplicon by standard techniques. After extensive washing, filters were exposed to X-ray films and the intensity of the bands quantified by Instant Imager (Packard Instruments, Downers, IL, USA).

### 2.7. Statistical Methods

Analysis of the results from animal studies was performed according the test on equality of proportion by STATA program. Statistical evaluation of the *in vitro* data was performed by the analysis of variance model (ANOVA) with Tukey-Kramer adjustment for multiple comparisons, using the SAS software, version 9.1 (SAS Institute, Cary, NC, USA). A *P*-value of <0.05 was considered as significant. 

## 3. Results

### 3.1. Induction of Angioproliferative Lesions by FGF-2 Is Associated with Bcl-2 Expression, and Both Are Enhanced by Tat

Initial experiments investigated FGF-2 or Tat effects on Bcl-2 expression *in vivo*. 

Our previous work tested the dose response effects that FGF-2 or Tat (0.1–100 *μ*g) has on the induction of histological features characterizing KS lesions *in vivo* [3, 4, 24]. Starting from those findings, we employed the Tat and/or FGF-2 concentrations particularly effective at promoting the development of macroscopic KS-like lesions in mice.

Specifically, nude mice were injected with 1 *μ*g of FGF-2 and/or 10 *μ*g of Tat. Control animals were inoculated with the protein suspension buffer (PBS-0.1% BSA). After 7 days, mice were sacrificed and the sites of injection were examined for the presence of angioproliferative lesions [3, 4]. Tissue samples were excised and processed for histology and Bcl-2 immuno-histochemistry. 

**Figure 1:**
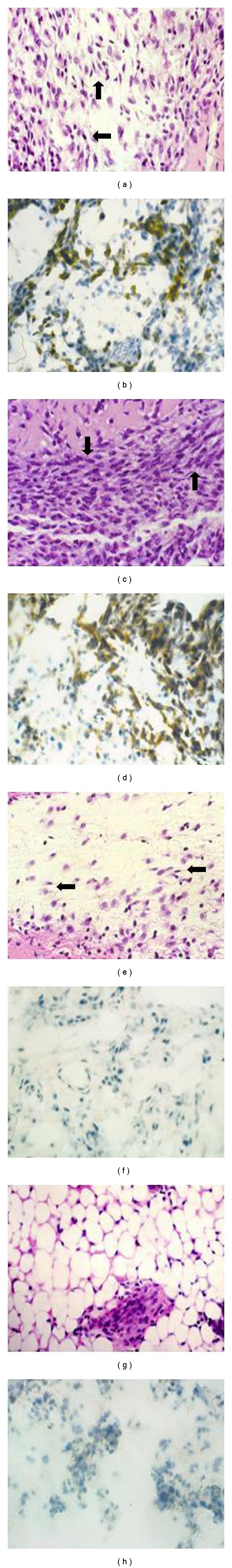
Induction of Bcl-2 expression in mice by FGF-2 and Tat. Mice were injected with 1 *μ*g of FGF-2 and/or 10 *μ*g of Tat, as described in Section 2. After 7 days tissue samples were taken at the injection sites and processed for histological examination by hematoxylin-eosin staining (left panels, 40X magnification). Tissue sections from 12 animals per group were stained with anti-Bcl-2 antibody (right panels, 40X magnification). Induction of KS-like lesions promoted by 1 *μ*g FGF-2 (a) was associated with Bcl-2 protein expression (b). The simultaneous inoculation of 1 *μ*g FGF-2 and 10 *μ*g Tat further increased both lesion development (c) and Bcl-2 expression (d). The inoculation of 10 *μ*g Tat alone induced only limited histological alterations (e) and no Bcl-2 protein expression (f) at the injection sites. Protein suspension buffer (PBS-0.1% BSA) did not promote lesion development (g), nor Bcl-2 protein expression (h). Arrows point at some representative spindle cells of KS-like lesions.

As shown in Figure 1, FGF-2 injection resulted in the development of macroscopic angioproliferative KS-like lesions. Specifically, 19 out of the 43 mice injected with FGF-2 ( = 44%,  *P* < 0.001) developed KS-like lesions. This was associated with the appearance of spindle-shaped cells (Figure 1(a)), which are the histological hallmark of human KS lesions [1], and with the induction of Bcl-2 expression (Figure 1(b)). In particular, 32% (range 19–49) of the cells present in the lesions were positive for Bcl-2. 

Noteworthy, the simultaneous inoculation of FGF-2 and Tat further increased both lesion development and Bcl-2 expression. In particular, out of the 31 mice injected with combined FGF-2 and Tat, 22 (=71%, *P* < 0.05) developed macroscopic lesions (Figure 1(c)), in which 47% (range 22–67) of the cells were Bcl-2 positive (Figure 1(d)).

In contrast, none of the 17 mice injected with Tat alone, and none of the 70 mice injected with the protein suspension buffer (PBS-0.1% BSA), developed macroscopic lesions (Figures 1(e) and 1(g), resp.). For both experimental conditions, Bcl-2 was not expressed at the sites of injection (Figures 1(f) and 1(h), resp.).

**Figure 2:**
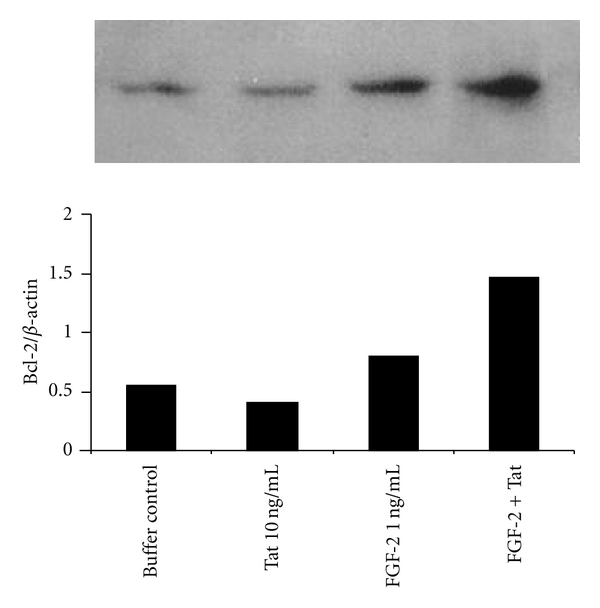
Induction of Bcl-2 protein expression by FGF-2 and/or Tat in human primary endothelial cells. HUVECs were cultured in the presence of Tat (10 ng/mL) and/or FGF-2 (1 ng/mL). HUVECs treated with the protein suspension buffer (PBS-0.1% BSA) were used as controls. After 24 hours of culture, HUVECs were lysed, and equal amounts of total proteins were electrophoresed and analysed by Western blotting using monoclonal antibodies directed against human Bcl-2. Blots were reprobed with anti-*β* actin monoclonal antibodies to verify equal loading of protein in each lane. In the upper panel a representative Western blot for Bcl-2 protein is shown. In the lower panel is its densitometric analysis, which is presented as the Bcl-2 to *β* actin ratio. Repeated experiments (two) gave similar results.

To confirm these *in vivo* results, the effects of FGF-2 and Tat on Bcl-2 expression were evaluated in cultured human primary endothelial cells. To this end, HUVECs were exposed for 24 hours to FGF-2 and/or Tat (controlled by their suspension buffer), and Bcl-2 protein levels were examined by Western blot analysis. As shown in Figure 2, FGF-2 increased Bcl-2 protein expression, while Tat alone had no effect. However, when Tat was combined with FGF-2, Bcl-2 protein levels further increased, confirming the *in vivo* data.

### 3.2. FGF-2 and Tat Protect Nonadherent Endothelial Cells from Apoptosis via the Upregulation of Bcl-2 Expression

Bcl-2 can protect endothelial cells from anoikis, the process where adherent cells die when they lose contact with the ECM [25, 26].

In view of our *in vivo* and *in vitro* results, additional work was performed in order to evaluate FGF-2 or Tat effect on endothelial cell anoikis. To this end we seeded HUVECs onto plates coated with poly- (HEMA), a compound preventing cellular adhesion [23]. Then, we incubated the cells for 6 h with FGF-2, Tat, or their suspension buffer. The entity of cell death was determined by measuring, in HUVECs cytoplasm, the content of nucleosomes, histone complex of low-molecular-weight DNA fragments resulting from the cleavage of apoptotic cell genome [27]. Adherent HUVECs plated onto gelatin were the negative control for cell death [25, 26].

**Figure 3:**
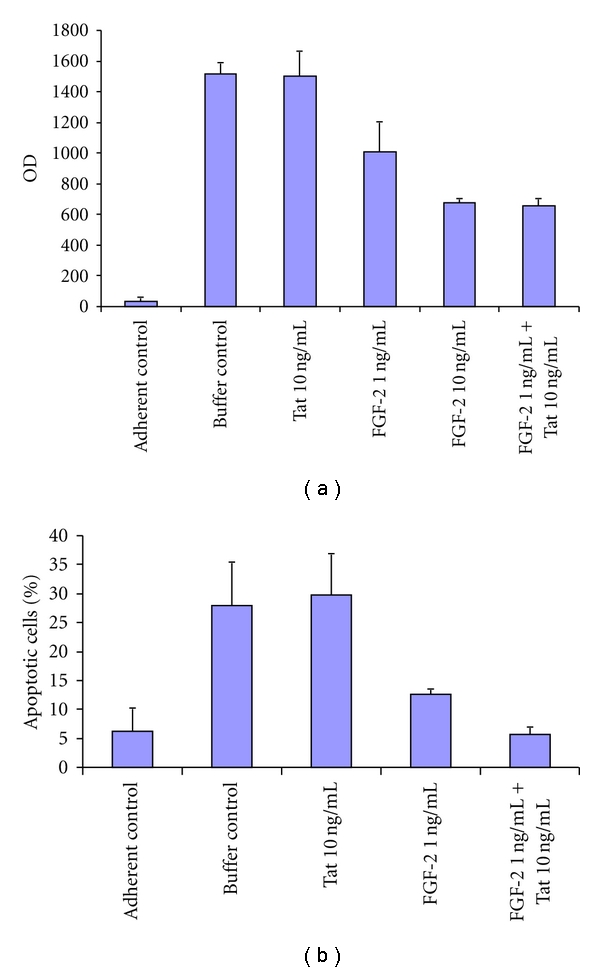
Effect of FGF-2 and Tat on human endothelial cell anoikis. HUVECs were seeded onto poly- (HEMA)-coated plates and incubated for 6 h with FGF-2 (1 or 10 ng/mL) and/or Tat (10 ng/mL), controlled by their dilution buffer (PBS-0.1% BSA). HUVECs plated onto gelatin were the negative control for apoptosis. In (a) are the results from the ELISA test determining the amount of nucleosomes in HUVECs cytoplasm. The mean OD readings from 4 independent experiments (+SD) are presented. In (b) HUVECs apoptosis was evaluated by TUNEL test. The mean percentage (+SD) of apoptotic cells was determined from 4 high-power fields (40X magnification) of 3 independent experiments.

As expected, HUVECs did not adhere onto poly- (HEMA)-coated surfaces, and this was followed by a dramatic increase of nucleosome content in the cytoplasm of nonadherent, as compared to adherent, HUVECs (*P* < 0.0001) (Figure 3(a)). Noteworthy, exposure of nonadherent HUVECs to 1 or 10 ng/mL FGF-2 reduced the amount of cytoplasmic nucleosomes by 30% and 50%, respectively (*P* < 0.0001) (Figure 3(a)). Although Tat alone had no effect, when it was combined with 1 ng/mL of FGF-2, it decreased the amount of cytoplasmic nucleosomes to levels as found in HUVECs treated with 10 ng/mL FGF-2 (Figure 3(a)). Thus, Tat increased FGF-2 capability of rescuing endothelial cell apoptosis  (*P* = 0.0102).

The TUNEL assay confirmed these results. Specifically, in nonadherent conditions, 1 ng/mL of FGF-2 diminished the number of apoptotic HUVECs by 55% when employed alone (*P* = 0.0490) and by 80% when combined with 10 ng/mL of Tat (*P* = 0.0025) (Figure 3(b)). 

To investigate whether the increase of endothelial cell survival promoted by FGF-2 and Tat in nonadherent conditions was accompanied by a modulation of Bcl-2 expression, Bcl-2 mRNA levels were evaluated in HUVECs under the different experimental conditions described above. 

**Figure 4:**
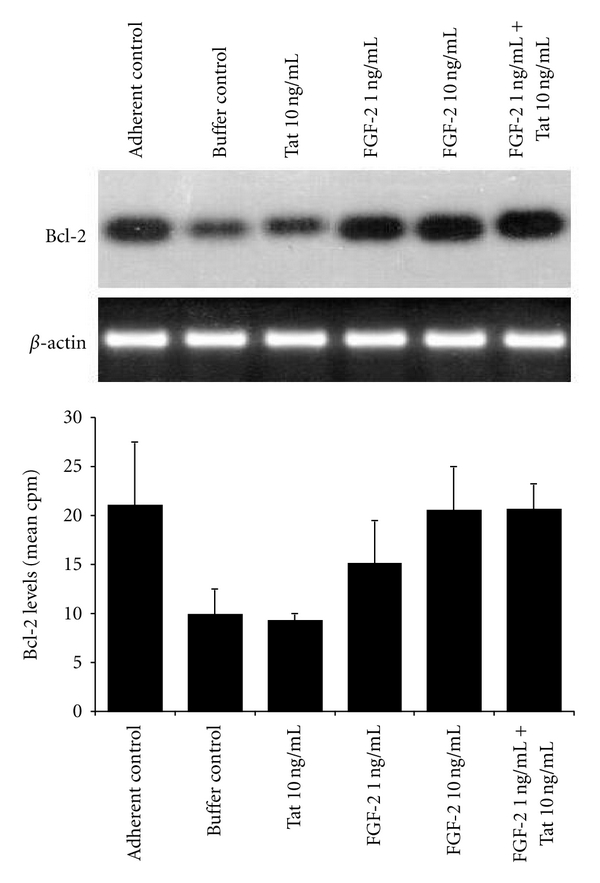
Protection of human endothelial cells from anoikis by FGF-2 alone or combined with Tat parallels the up-regulation of Bcl-2 expression. HUVECs were treated as described in the legend of Figure 2. Total RNA was extracted from the cells, and the Bcl-2 mRNA levels were analyzed by a housekeeping gene normalized RT-PCR assay. In the upper panel is the Southern blot hybridization of PCR products from a representative RT-PCR experiment performed with primers specific for Bcl-2. In the central panel is the *β*-actin RT-PCR amplification of the samples, showing that normalization of cDNA content resulted in *β*-actin PCR bands of identical size. In the lower panel is the quantification of Bcl-2 hybridization bands by Instant Imager from 3 independent experiments (average +SD).

Results from RT-PCR assays indicated that Bcl-2 mRNA levels decreased by −53% in nonadherent HUVECs, as compared to the adherent control (*P* = 0.0396) (Figure 4). When nonadherent HUVECs were exposed to 10 ng/mL of FGF-2, Bcl-2 mRNA expression was rescued to levels as expressed by adherent HUVECs (*P* = 0.05) (Figure 4). Although Tat alone had no effect, when it was combined with 1 ng/mL of FGF-2, the expression of Bcl-2 was restored to the levels detected in adherent HUVECs (*P* = 0.0480) (Figure 4). 

## 4. Discussion

In healthy tissues, undesired angiogenesis is switched off through the induction of endothelial cell apoptosis [25, 28]. This can be triggered by conditions, such as the lack of angiogenic growth factors, or the inhibition of endothelial cell adhesion onto the ECM, which downregulate endothelial cell expression of antiapoptotic molecules [25, 28].

Among the latter, Bcl-2 is very effective at favouring the angiogenic process. In particular, Bcl-2 is overexpressed by endothelial cells of the newly formed blood vessels nourishing the growing tumor [29, 30].

In agreement with the fact that Bcl-2 expression can lead to inappropriate endothelial cell survival and abnormal angiogenesis [25, 28], clinical evidence suggests that the deregulation of Bcl-2 expression could play a role in the maintenance and progression of KS. In fact, Bcl-2 protein levels augment in late-stage, as compared to early-stage, KS lesions, and this parallels a decrease in vascular cell apoptosis [8–12]. 

A role for Bcl-2 in KS progression is supported also by the efficacy of the Bcl-2 inhibitor paclitaxel in KS patients [31], confirming previous work performed with animal models of KS [32]. 

Here, we evaluated whether FGF-2 or HIV-1 Tat, two key AIDS-KS pathogenetic factors, could affect Bcl-2 expression *in vivo* and *in vitro*. 

Results indicate that FGF-2 injection into mice results in the induction of Bcl-2 expression and that this associates with the development of angioproliferative, KS-like lesions. 

Regarding Tat, it has no effects on Bcl-2 expression when present alone. Tat protein is known to enter cells and activate the expression of cellular genes [6]. In particular, Tat was shown to up-regulate Bcl-2 expression by monocytes, albeit at higher concentrations than the ones we used here [33]. However, the possibility that in our experimental model Tat could directly activate Bcl-2 expression is excluded by our results indicating that Tat alone is not capable of promoting KS-like lesions in mice, rescuing endothelial cell apoptosis or inducing Bcl-2 expression by these cells. The contrasting data that others obtained in monocytes could depend on the different cell type they utilized. The fact that Tat effects are cell-type dependent is strongly supported by previous findings indicating that Tat promotes apoptosis in other cell systems [34–39].

In contrast, when Tat is combined with FGF-2, it strongly enhances FGF-2 effects on Bcl-2, this paralleling a dramatic increase in the formation of angioproliferative lesions in mice.

These findings are consistent with all the other activities that Tat exerts on endothelial cells, which require FGF-2 presence in order to respond to Tat either *in vitro* or *in vivo *[3, 4, 7]. In this regard, Tat and FGF-2 synergy is likely to depend on FGF-2 capability of inducing the expression of the *α*_*v*_ *β*_3  _and *α*_5_ *β*_1  _integrins, which, in turn, function as Tat receptors [3, 4, 7, 40]. On the other side, Tat can maintain FGF-2 into a highly active and readily available form, thus amplifying its biological effects [7]. 

Previous studies indicated that the cross-talk between the integrins and growth factor receptors triggers a signalling cascade which is relevant for endothelial cell survival and growth [25, 28]. In this regard, it has to be reminded that signalling pathways leading to endothelial cell survival are activated upon Tat binding to the integrins, being further enhanced when FGF-2 is present in the environment [41]. In this context, our previous work indicated that induction of KS-like lesions by combined FGF-2 and Tat is reproduced by combined FGF-2 and the integrin-binding Tat RGD peptide (Tat 65–80), but not by mutated Tat KGE peptide [4].

In agreement with these findings, here we have reported that the induction of Bcl-2 expression by FGF-2 and Tat can prevent the death of endothelial cells resulting from the inhibition of their adhesive interaction with the ECM. 

Altogether, these results suggest a pathway for Bcl-2 induction and a molecular mechanism by which the simultaneous presence of Tat and FGF-2 could promote KS maintenance and progression in HIV-1-infected individuals. Since Tat and FGF-2 are expressed in AIDS-KS lesions together with Bcl-2 [1, 3], these findings appear to be of biological relevance. 

Finally, as Bcl-2 diverts vascular cells from apoptosis and prolongs their survival [21, 29, 30, 42], its expression in advanced KS, coupled with a deregulated cellular proliferation by HIV, HHV8, and cellular factors [1, 2], may result in KS maintenance and/or the progression of the early angio-hyperplastic KS lesions into a true sarcoma. This hypothesis is supported by our recent observation that both FGF-2 and Tat can increase in KS cells the activity of the telomerase, an enzyme allowing cells to escape replicative senescence and to proliferate indefinitely [43, 44].

## 5. Conclusions

Our results indicate that FGF-2 and HIV-1 Tat synergize at inducing Bcl-2 expression and that this is accompanied by the inappropriate survival of endothelial cells and by the development of angioproliferative lesions. These findings suggest a molecular mechanism for the deregulated Bcl-2 expression observed in AIDS-KS lesions and confirm that Bcl-2 may represent a key target for therapeutic intervention of KS.
